# Environmental and social-demographic predictors of the southern house mosquito *Culex quinquefasciatus* in New Orleans, Louisiana

**DOI:** 10.1186/s13071-018-2833-5

**Published:** 2018-04-17

**Authors:** Imelda K. Moise, Claudia Riegel, Ephantus J. Muturi

**Affiliations:** 1 Department of Geography and Regional Studies, College of Arts and Sciences, University of Miami, 1300 Campo Sano Ave, Coral Gables, FL 33124 USA; 2 Department of Public Health Sciences, Miller School of Medicine, University of Miami, 1120 NW 14th Street, Miami, FL 33136 USA; 3 New Orleans Mosquito, Termite and Rodent Control Board, 2100 Leon C. Simon, New Orleans, LA 70122 USA; 4 Crop Bioprotection Research Unit, USDA, ARS, 1815 N. University St, Peoria, IL 61604 USA

**Keywords:** GIS, Remote sensing, New Orleans, Mosquitoes, Hurricane Katrina

## Abstract

**Background:**

Understanding the major predictors of disease vectors such as mosquitoes can guide the development of effective and timely strategies for mitigating vector-borne disease outbreaks. This study examined the influence of selected environmental, weather and sociodemographic factors on the spatial and temporal distribution of the southern house mosquito *Culex quinquefasciatus* Say in New Orleans, Louisiana, USA.

**Methods:**

Adult mosquitoes were collected over a 4-year period (2006, 2008, 2009 and 2010) using CDC gravid traps. Socio-demographic predictors were obtained from the United States Census Bureau, 2005–2009 American Community Survey and the City of New Orleans Department of Code Enforcement. Linear mixed effects models and ERDAS image processing software were used for statistical analysis and image processing.

**Results:**

Only two of the 22 predictors examined were significant predictors of *Cx. quinquefasciatus* abundance. Mean temperature during the week of mosquito collection was positively associated with *Cx. quinquefasciatus* abundance while developed high intensity areas were negatively associated with *Cx. quinquefasciatus* abundance.

**Conclusion:**

The findings of this study illustrate the power and utility of integrating biophysical and sociodemographic data using GIS analysis to identify the biophysical and sociodemographic processes that increase the risk of vector mosquito abundance. This knowledge can inform development of accurate predictive models that ensure timely implementation of mosquito control interventions.

## Background

Mosquitoes pose serious economic and public health challenges due to their role in transmission of debilitating and life-threatening diseases of human, veterinary and wildlife significance [1]. These include parasitic diseases such as malaria and lymphatic filariasis and arthropod-borne viruses (arboviruses) such as dengue, chikungunya, Zika and West Nile virus (WNV). Transmission of these diseases follow a clearly defined spatial and temporal pattern that is closely associated with mosquito distribution, abundance and vectorial capacity [2, 3]. Each mosquito species has specific biological and ecological requirements that dictate their occurrence, distribution, abundance and vectorial capacity. These requirements encompass a complex network of factors related to climate regimes (e.g. temperature and rainfall), land use, land cover, topography and socioeconomic factors [4–8]. The number and type of factors that influence mosquito distribution can vary markedly between geographical locations even for the same mosquito species [9]. Therefore, identifying the local climatic, environmental, and socioeconomic factors that influence mosquito distribution and abundance can facilitate prediction of disease transmission cycles and timely implementation of targeted surveillance and intervention measures.

Natural disasters such as flooding can have major impacts on mosquito-borne disease transmission by modifying the climatic, environmental and socioeconomic drivers of mosquito distribution and abundance. Hurricane Katrina hit the city of New Orleans on August 25, 2005 and presented one of the recent examples of how natural disasters can influence mosquito population dynamics and mosquito-borne disease transmission risk. Levee failures associated with Hurricane Katrina led to massive flooding where approximately 80% of the city of New Orleans was flooded. These floods created numerous water bodies including abandoned swimming pools that were associated with large populations of mosquito larvae including *Culex quinquefasciatus* Say [10, 11], the primary vector of WNV in Louisiana and neighboring states [12]. The observed increase in mosquito larval populations coincided with warm and wet weather, which favored mosquito production [13]. Further studies revealed a 2-fold increase in the number of reported cases of West Nile neuroinvasive disease (WNND) in the hurricane-affected regions of Louisiana and Mississippi compared to previous years [14]. However, there has been no comprehensive studies to identify the major predictors of mosquito abundance in New Orleans post Katrina.

Mosquito-borne disease risk in the aftermath of disasters is likely to vary across space, such that high and low levels of risk are concentrated in specific geographical areas. In addition, the extent to which climatic, neighborhood and environmental characteristics are associated with post-disaster mosquito activities is likely to vary across geographical regions. Therefore, there is potential to use modern scientific geospatial data analysis to integrate both spatial and non-spatial data seamlessly [15] to better understand the spatial and temporal distribution of mosquito vectors in flood prone areas. Moreover, extensive research has shown that when coupled with traditional vector surveillance and environmental monitoring [16, 17], geospatial techniques can help in examining changes in mosquito densities and allow for the identification of climatic, neighborhood and environmental predictors that may be associated with high vector densities [10, 18–24].

The current study uses different geospatial tools to perform a comprehensive analysis aimed at identifying the primary climatic, environmental and sociodemographic predictors of the southern house mosquito *Cx. quinquefasciatus* in New Orleans, Louisiana, USA. The findings of this study are crucial for the identification of target hotspots where limited human and fiscal resources can be directed for effective and timely control of vector mosquitoes and prevention and management of disease outbreaks [4, 5].

## Methods

### Study area

New Orleans is located in southeastern Louisiana, straddling the Mississippi River, between longitude 90°4'14"W and latitude 29°57'53"N. The lowest point in Louisiana is 8 feet "below" sea level and is located in New Orleans Parish (Orleans Parish and the City of New Orleans are coterminous). The average annual rainfall for the City of New Orleans from 1981 to 2010 is 62.3 inches (2.88 inches more than the average in Louisiana) and the maximum and minimum average monthly temperatures are 90.6 °F and 41.8 °F, respectively. Peak rainfall generally occurs from June through September. Before Hurricane Katrina, Orleans Parish had 437,186 inhabitants and dealt with mosquito-transmitted viral diseases since its inception as a French colonial city in 1718. Fortunately, there have been relatively few human cases of WNV, with only 10 reported cases of WNV in 2002 and 11 cases reported in 2011 [25]. The reported cases occurred during the months of July, August and September, the expected high-transmission months.

### Mosquito data

We obtained adult mosquitoes data from the City of New Orleans Mosquito, Termite and Rodent Control Board (NOMTCB). NOMTCB established permanent traps sparsely across New Orleans in the aftermath of Hurricane Katrina in the proximity of habitats producing biting nuisance and disease mosquito. In New Orleans, those areas included low- and medium-developed areas, inner marshes, saltwater or brackish tidal wetlands and the adjacent forested areas containing freshwater wetlands. Notably, NOMTCB has expanded its mosquito surveillance operations since 2010. Hence, the trap locations used in the current study represented areas that experienced Hurricane Katrina’s disastrous flooding on August 29, 2005 and remained representative of the different patterns of land use and land cover changes that have occurred since Hurricane Katrina.

Nineteen CDC gravid traps (John W. Hock Company, Gainesville, FL, USA) and Frommer updraft gravid trap (John W. Hock Company, Gainesville, FL, USA) baited with fish-oil emulsion were used to collect adult mosquito data. The traps operated from March through September during years 2006, 2008, 2009 and 2010. Mosquito capture data were retrieved 3–4 times per week, brought to the NOMTCB laboratory and identified by trained entomologists under a microscope using morphological taxonomic keys of Darsie & Ward [26]. Nineteen permanent traps were used in the analysis with the annual summaries obtained for years 2008, 2009 and 2010.

### Mosquito data processing

Trapping effort was relatively regular from the three years under study. To avoid issues of spatial autocorrelation in abundance, all mosquito capture data at each trap site were calculated as weighted average of a one-kilometer buffer around each trap (based on average effective flight range of the *Cx. quinquefasciatus*), monthly and annually.

### Environmental data collection and processing

To quantify land use and land cover (LULC), we derived Landsat 7 EMT (Enhanced Thematic Mapper) satellite imagery patch/row 22/39 (essentially cloud-free) covering New Orleans (dated September 5, 2010) with a ground resolution of 30 × 30 m. The data were processed using ERDAS (2002) image processing software. Supervised classification was performed to cluster pixels in the subset image data into land cover classes. This was done by defining regions of interest (ROI) that represented each of the four desired LULC classes in the output image corresponding to possible adult *Cx. quinquefasciatus* habitats in New Orleans. The ROI’s included: developed, open space (mostly vegetation in the form of lawn grasses); developed low intensity (mostly areas with a mixture of constructed materials and vegetation); developed medium intensity (mostly single-family housing units); developed high intensity (highly developed areas where people reside or work in high numbers); open water (areas of open water); and woody wetlands (see National Land Cover Database 2006, NLCD2006; http://www.mrlc.gov/nlcd06_leg.php for class definitions). Utmost attention was made in selecting ROI that are homogeneous and correcting for overlaps between classes. We then performed maximum likelihood classification to assign each pixel in the subset image data to the class that had the highest probability.

To derive land cover composition values around each trap location, a one-kilometer buffer was created around each trap (based on average effective flight range of the vector) for each of the three years with observed peak capture (2008, 2009 and 2010) covering the 19 traps under study. We used this buffer to characterize the environment that mosquitoes inhabited around the trap location, considering that in an urban environment, movement is localized due to abundant proximate resources (e.g. blood hosts and container habitats).

### Weather data

Historical monthly temperature and precipitation were obtained from the Louis Armstrong New Orleans International Airport (KMSY) located 10 nautical miles (19 km) west of New Orleans’ central business district. Although New Orleans has two weather stations, only KMSY weather station data is used since it possessed comprehensive meteorological data. The data included daily maximum temperature, daily minimum temperature and daily total precipitation, which the other weather station did not have.

### Sociodemographic data

We obtained neighborhood and socio-demographic data from the City of New Orleans Department of Code Enforcement (http://www.nola.gov/). Predictors included a list of address-level data on blighted properties [completed property demolition listings (2006–2009), proposed imminent health threat listed properties (2009) and danger of collapse or public nuisance listed properties (2009)]. From the NOMTCB we also obtained data on abandoned swimming pools [22]. Socioeconomic data were obtained from the U.S. Census Bureau, 2005–2009 American Community Survey (ACS). These predictors included population density, change in number of households and median household income.

We then performed an area-weighted average to measure climate, land use, land cover and socio-demographic predictors for the area covered by each of the trap buffers during the three years (2008, 2009 and 2010 - three years with comparable trap data). Gravid trap characterization was computed using Imagine Analysis in ArcGIS, version 10.3 [25].

### Statistical analyses

Statistical analyses were conducted using R version 3.3.3 software [27]. Only female *Cx. quinquefasciatus* were included in the analyses because the abundance of the other mosquito species was too low. Mosquito data were log10 (x + 1) transformed to meet the assumptions of normality. For both temperature and precipitation data, we calculated the average for each week as well as the average for 1 week (Lag 1) and 2 weeks (Lag 2) prior to mosquito collection in order to take into account the cumulative effect of each variable (rainfall and temperature) on mosquito production and abundance [28].

With the additional rainfall and temperature variables (1-week lag and 2-week lag), a total of 22 continuous variables were generated. To test for collinearity and remedy the situation, a linear regression model was fitted to assess the relationship between mosquito abundance and the 22 continuous variables. The package “*car*” was used to identify and remove highly correlated variables through sequential removal of all variables with a variance inflation factor (VIF) greater than 2.5. Seven variables that met this criterion were used for subsequent analysis. These included variables derived from land cover composition values areas of open water, developed low intensity, developed high intensity and developed open space; weather variable mean temperature (°F); neighborhood recovery variable unattended swimming pools, and sociodemographic variable population density.

The package “*Boruti*” was used to check which of the 7 retained variables were important and the following six variables were confirmed to be important: mean temperature (°F), mean temperature at lag 2 (two weeks prior to mosquito collection), mean precipitation at lag 1, developed, open space, developed high intensity and developed grassland (e.g. golf courses). These six continuous independent variables were analyzed as fixed factors while year was used as a random variable in a linear mixed effects model conducted using “*lme4*” package.

## Results

### Mosquito collection

**Fig. 1:**
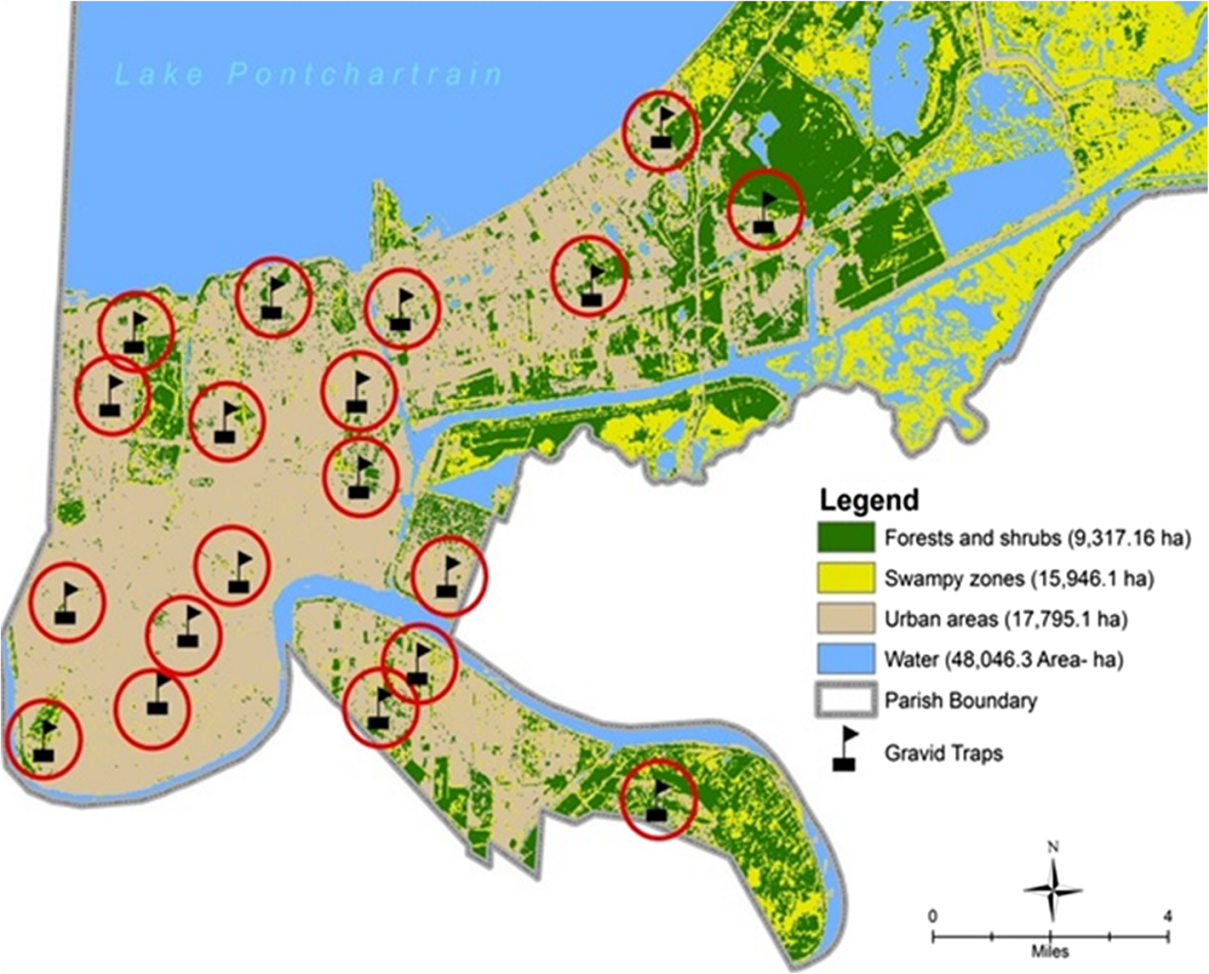
Nineteen trap locations where adult mosquitoes were collected from March 2009 to September 2010. The 1-km radius surrounding each trap used in analysis is outlined in red. Traps are overlaid on a land cover/land use map

From 2008 to 2010, 72,082 adult female mosquitoes belonging to three mosquito species were collected from 19 gravid traps across New Orleans (Table 1). These included *Aedes aegypti* (Linnaeus in Hasselquist), *Aedes albopictus* (Skuse) and *Cx. quinquefasciatus*, with the *Cx. quinquefasciatus* accounting for 82.4% of the total collection. The mean annual abundance of *Cx. quinquefasciatus* was summarized across all 19 traps (Fig. 1).

**Table 1:** Number of mosquito species collected in each genus by study site in New Orleans summarized by month from spring 2006 to summer 2010

5.Year	Month	No. of collections	*AEG n* (%)	*ALB n* (%)	*CXQ n* (%)	Total no. of mosquitoes
2006	March	30	0 (0.00)	18 (0.04)	46 (0.06)	64
2006	April	29	1 (0.16)	2 (0.00)	40 (0.06)	43
2006	May	34	38 (5.89)	12 (0.03)	164 (0.23)	214
2006	June	27	29 (4.50)	2 (0.00)	316 (0.44)	347
2006	July	34	31 (4.81)	ns	291 (0.41)	322
2006	August	54	105 (16.28)	5 (0.01)	343 (0.48)	453
2006	September	44	36 (5.58)	3 (0.01)	228 (0.32)	267
2008	March	4	0 (0.00)	0 (0.00)	2 (0.00)	2
2008	April	56	8 (1.24)	3 (0.01)	634 (0.89)	645
2008	May	55	9 (1.40)	7 (0.02)	1592 (2.24)	1608
2008	June	56	26 (4.03)	10 (0.02)	2558 (3.60)	2594
2008	July	70	50 (7.73)	30 (0.07)	3154 (4.44)	3234
2008	August	58	43 (6.67)	13 (0.03)	1493 (2.10)	1549
2009	March	54	1 (0.16)	2 (0.00)	881(1.24)	884
2009	April	48	6 (0.93)	2 (0.00)	2847 (3.60)	2,855
2009	May	67	19 (2.95)	16 (0.04)	12,646 (17.81)	12,681
2009	June	75	16 (2.48)	11(0.03)	5772 (8.13)	5799
2009	July	53	18 (2.79)	51(0.12)	2354 (3.31)	2423
2009	August	58	54 (8.37)	56 (0.13)	3501 (4.93)	3611
2009	September	63	55 (8.37)	43 (0.41)	5666 (7.98)	5764
2010	March	38	0 (0.00)	0 (0.00)	5 (0.01)	5
2010	April	38	0 (0.00)	3 (0.01)	380 (0.54)	383
2010	May	75	9 (1.10)	29 (0.07)	9384 (13.21)	9422
2010	June	71	41 (6.36)	20 (0.05)	8894 (12.52)	8955
2010	July	52	18 (2.79)	47 (0.11)	4867 (6.85)	4932
2010	August	91	28 (4.34)	30 (0.07)	2841 (4.00)	2899
2010	September	18	4 (0.62)	5 (0.01)	118 (0.17)	127
Total			645	420	71,017	72,082

*Abbreviations*: *CXQ Culex quinquefasciatus*, *AEG Ae. aegypti*, *ALB Ae. albopictus*, *ns* not sampled

### Mosquito abundance change over time

**Fig. 2:**
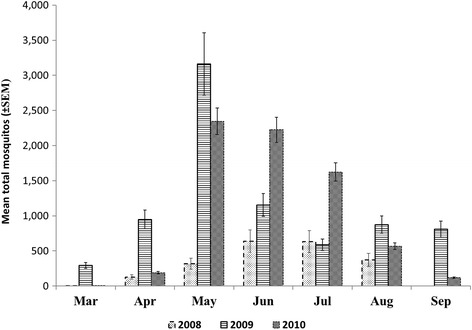
Monthly variation in *Cx. quinquefasciatus* abundance

There was significant temporal (ANOVA: *F* = 5.922, *df* = 2.51, 100, *P* < 0.002) and inter-annual variation in the abundance of *Cx. quinquefasciatus* (Fig. 2). Significantly more mosquitoes were collected in 2009 (*n* = 33,667) and 2010 (*n* = 26,489) compared to 2008 (*n* = 9431) (*P* < 0.01). Peak mosquito populations also occurred much earlier in 2009 and 2010 (May) compared to 2008 (July). This suggests that the peak extended over June-July in 2008 and in 2010 (May-June).

### Association of LULC, climate and socio-demographic predictors with mosquito abundance

The random variable year accounted for 4.0% of the total observed variance suggesting it had little influence on adult mosquito abundance. Mosquito abundance was positively associated with mean temperature of the week when the mosquitoes were collected and negatively associated with highly developed areas, in particular areas where people reside or work in high numbers (Table 2). None of the other variables was significantly associated with mosquito abundance.

**Table 2:** LULC, weather and sociodemographic predictors of *Cx. quinquefasciatus* abundance, New Orleans, 2009 and 2010

	Estimate	SE	*t*-value	Chi-square	*P*-value
(Intercept)	1.08	0.12	9.08		
Mean temperature (°F)	0.17	0.04	4.28	18.29	< 0.001
Lag 1 mean temperature (°F)	-0.03	0.04	-0.82	0.68	0.41
Lag 1 mean precipitation	0.05	0.03	1.70	2.88	0.09
Lag 2 mean temperature (°F)	0.00	0.04	0.04	0.00	0.97
Developed, open space areas	0.00	0.03	0.09	0.01	0.93
Developed high intensity areas	-0.18	0.03	-5.66	32.06	< 0.001
Developed grassland areas	0.05	0.03	1.63	2.66	0.10

*Abbreviation*: *SE* standard error

## Discussion

The aim of this study was to examine the environmental, climatic and sociodemographic factors that influence the spatial and temporal distribution of *Cx. quinquefasciatus* abundance in New Orleans, Louisiana, from 2006 to 2010. Female host-seeking *Cx. quinquefasciatus* were captured with CDC gravid traps, so their abundances reflected the blood meal-seeking behavior of this species. Our findings show that mosquito abundance was low in 2008 and increased dramatically in 2009. In New Orleans, this finding may be attributable to the accelerated and targeted surveillance and control efforts that were implemented by NOMTCB after Hurricane Katrina, which may have contributed to the prolonged reduction in adult mosquito abundance [22].

Another important finding was that *Cx. quinquefasciatus* abundance was positively associated with mean temperature of the week when mosquitoes were collected and was negatively associated with highly developed areas. The former finding is concordant with previous findings [29], and other studies, which found temperature as influencing oviposition and habitant selection by *Cx. quinquefasciatus* [30, 31]. These results also seem to be consistent with other research, which found temperature to accelerate the development of mosquito larvae and higher mosquito abundance [32–36]. It can therefore, be assumed that our models depicted the positive impacts of temperature at a biologically probable time lag that could provide for a basis for planning mosquito control activities in New Orleans. This is important because *Cx. quinquefasciatus* plays a very important role in the enzootic cycle due to its susceptibility to viral infection from feeding on infected blood meal and feeding preference for mammals instead of birds in urban environments [37–40].

One unanticipated finding was the negative association with highly developed areas and *Cx. quinquefasciatus* abundance. This outcome is contrary to that of previous studies that linked highly developed areas with WNV in New York [41] but they are broadly consistent with earlier studies that found low mosquito densities in highly developed areas in urban settings [29, 30]. In addition, in New Orleans, *Cx. quinquefasciatus* has been reported to oviposit in different water sources and achieving high larval densities in water with high organic content (e.g. sewage treatment ponds, drains) and mucky waters including abandoned swimming pools [10, 11, 23, 42]. Therefore, this finding was unexpected and suggests that the observed lower *Cx. quinquefasciatus* abundance is likely the result of NOMTCB control interventions targeted at eliminating larval sources rather than prevailing unaltered levels [22]. This finding, while preliminary, supports the need for continued mosquito control efforts in areas where surveillance indicates presence and increased risk of mosquitoes. The potential impacts of NOMTCB control interventions on vector abundance requires further study.

With the exception of mean temperature and highly developed areas, environmental and sociodemographic factors were not associated with *Cx. quinquefasciatus* abundance in New Orleans*.* These findings could be reflective of our study setting, a city that augmented mosquito control and outreach activities in the aftermath of Hurricane Katrina. Furthermore, given the unique climate, land use and hydrology of New Orleans, the details of these environmental and sociodemographic relationships will probably differ from other areas of the United States. Further studies, which consider these variables in other southeastern United States, will need to be undertaken.

Methodologically, our findings also highlight the importance of beefing up traditional non-spatially explicit methods with geospatial analysis (e.g. GIS) and satellite data to integrate environmental parameters in analyzing the spatiotemporal distribution of mosquito population and the underlying predictors. These methods allowed both the integration of biophysical and sociodemographic data, with the potential to better guide mosquito surveillance and control interventions [21, 43–45].

This study had two major weaknesses that limited the scope of analyses. First, we did not have pre-Katrina data in order to be able to compare changes in mosquito abundance before, during and after the Hurricane Katrina. However, our data still provides important knowledge on the key environmental factors that influence the abundance of one of the primary vectors of WNV in southeastern United States. Second, our attempt to validate any form of model fitting was hindered by the limited number of traps used in the current study, with only 19 traps. Additional studies using a much larger sample size could be more revealing.

## Conclusions

We examined the spatial and temporal changes in *Cx. quinquefasciatus* abundance in New Orleans (2006, 2008, 2009 and 2010) and association with environmental and social demographic predictors. *Culex quinquefasciatus* abundance increased significantly and had earlier peaks in 2009 and 2010 than in 2008. High mosquito abundance was negatively associated with highly developed areas while mean temperature during the week of mosquito collection was positively associated with *Cx. quinquefasciatus* abundance. Findings illustrate the power and utility of integrating biophysical and sociodemographic data using GIS analysis to assess influences of biophysical and sociodemographic processes that generate heterogeneity in critical *Cx. quinquefasciatus* mosquito abundance that inform the development of accurate predictive models that ensure timely implementation of mosquito control interventions.
